# Supplementary material for: Protection against Glucolipotoxicity by High Density Lipoprotein in Human PANC-1 Hybrid 1.1B4 Pancreatic Beta Cells: The Role of microRNA
Source: Biology (Basel). 2021 Mar 13;10(3):218. doi: 10.3390/biology10030218 (PMC8000094; doi:10.3390/biology10030218)
Supplement: Supplementary file 1 [file biology-10-00218-s001.pdf]

biology

Gene Symbol	NCBI Reference Sequence	Forward primer sequence	Reverse primer sequence
ABCA1	NM_005502.4	GTCAGCTGCTGCTGGAAGT	CCACCAGGAAATCTTGAAGC
ABCG4	XM_024452141.1	GGTTCATGTCCCACGTGGTT	GCCGGTGTTGTTGAAGACCTT
ZNF367	NM_153695.4	GGACAGCTCAAAACACATCAGCG	TTCGGACAGTGGCGGTTTGCAT
PDCD4	NM_014456.5	ACTGTGCCAACCAGTCCAAAGG	CCTCCACATCATACACCTGTCC
PPIA	NM_001300981	GGCAAATGCTGGACCCAACACA	TGCTGGTCTTGCCATTCCTGGA
STAT3	NM_139276.3	CTTTGAGACCGAGGTGTATCACC	GGTCAGCATGTTGTACCACAGG
FOXO3	NM_201559.3	TCTACGAGTGGATGGTGCGTTG	CTCTTGCCAGTTCCCTCATTCTG
SMAD7	NM_001190822.2	TGTCCAGATGCTGTGCCTTCCT	CTCGTCTTCTCCTCCCAGTATG
RPL13α	NM_012423.4	CTCAAGGTGTTTGACGGCATCC	TACTTCCAGCCAACCTCGTGAG

Table S1: Primer sequences for qPCR to measure mRNA expression of the genes specified.

Gene Symbol	NCBI Reference Sequence	Forward primer sequence	Reverse primer sequence
hsa-miR-21-5p	NR_029493.1	GCAGTAGCTTATCAGACTGATG	-
Universal Reverse Primer	-	-	GAATCGAGCACCAGTTACGC

Table S2: Primer sequences for qPCR to measure hsa-miR-21-5p expression.
